# Supplementary figures and images for: Greenhouse gas emissions from riparian zone cropland in a tributary bay of the Three Gorges Reservoir, China
Source: PeerJ. 2020 Feb 18;8:e8503. doi: 10.7717/peerj.8503 (PMC7034375; doi:10.7717/peerj.8503)

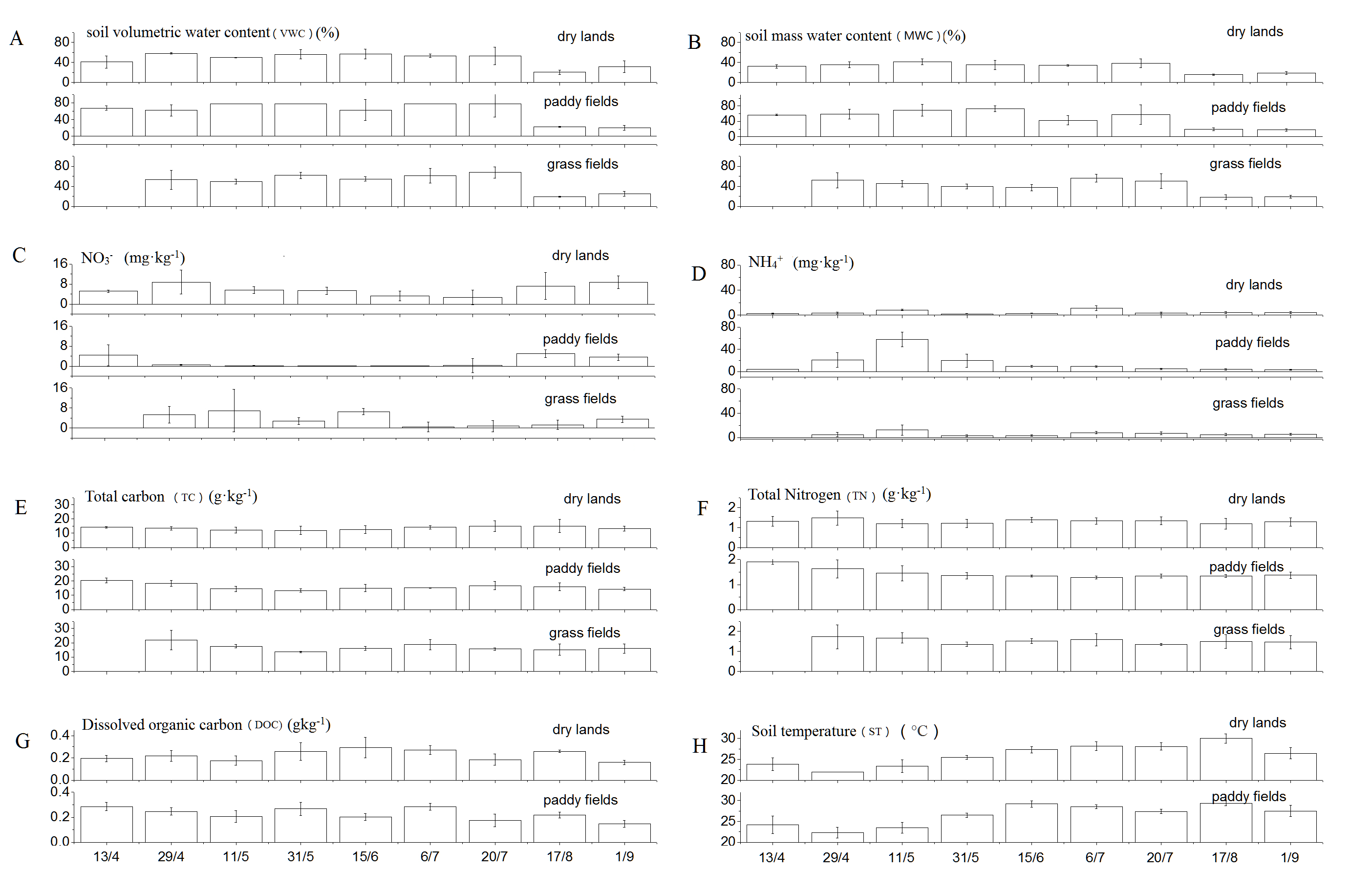

Supplement: Figure S1 [file peerj-08-8503-s001.png]

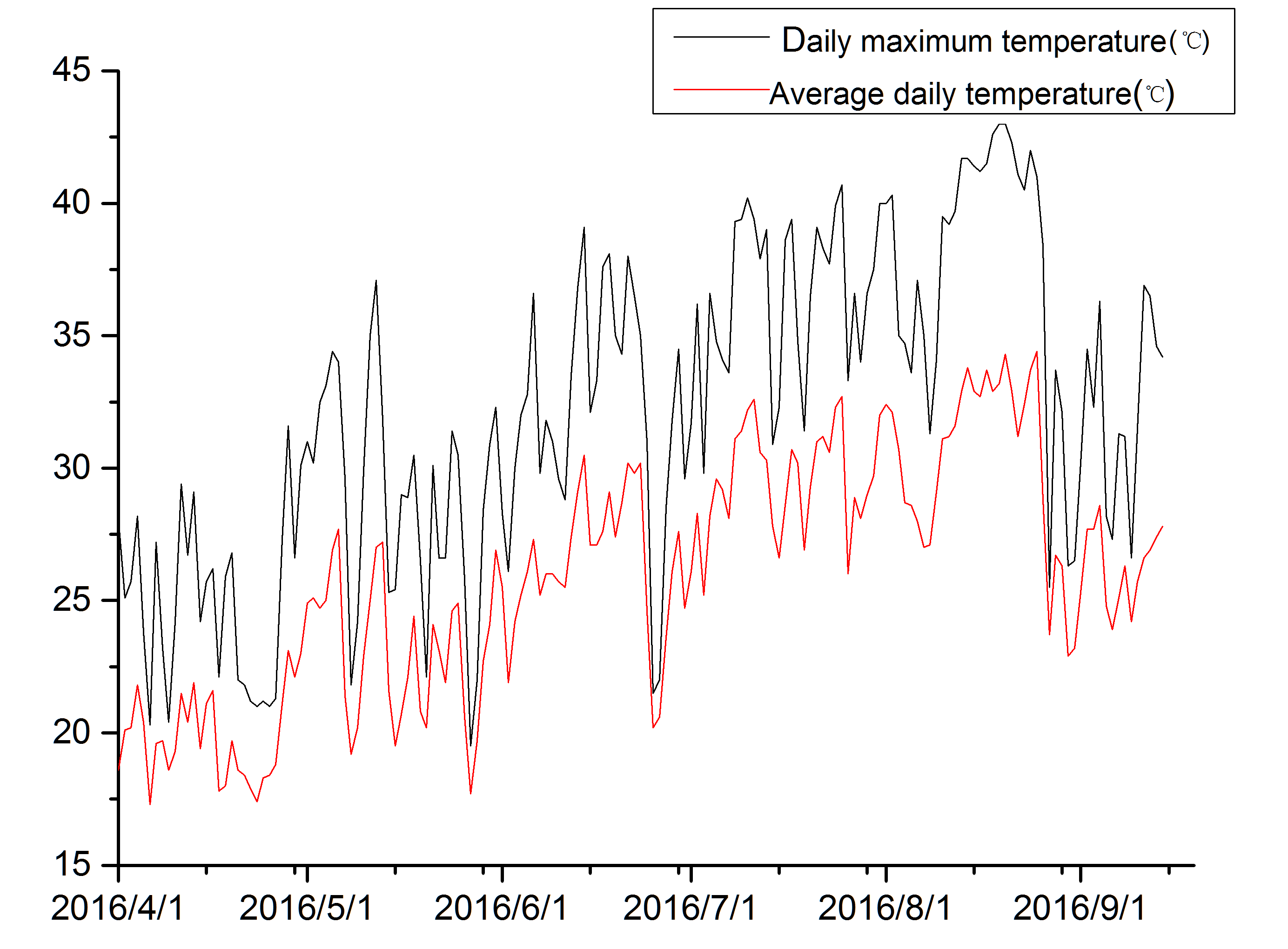

Supplement: Figure S2 [file peerj-08-8503-s002.png]
